# Engraftment of human induced pluripotent stem cell-derived hepatocytes in immunocompetent mice via 3D co-aggregation and encapsulation

**DOI:** 10.1038/srep16884

**Published:** 2015-11-23

**Authors:** Wei Song, Yen-Chun Lu, Angela S. Frankel, Duo An, Robert E. Schwartz, Minglin Ma

**Affiliations:** 1 Department of Biological and Environmental Engineering, Cornell University, Ithaca, NY 14850, USA; 2 Division of Gastroenterology & Hepatology, Department of Medicine, Weill Medical College of Cornell University, New York, NY 10021, USA

## Abstract

Cellular therapies for liver diseases and *in vitro* models for drug testing both require functional human hepatocytes (Hum-H), which have unfortunately been limited due to the paucity of donor liver tissues. Human pluripotent stem cells (hPSCs) represent a promising and potentially unlimited cell source to derive Hum-H. However, the hepatic functions of these hPSC-derived cells to date are not fully comparable to adult Hum-H and are more similar to fetal ones. In addition, it has been challenging to obtain functional hepatic engraftment of these cells with prior studies having been done in *immunocompromised* animals. In this report, we demonstrated successful engraftment of human induced pluripotent stem cell (iPSC)-derived hepatocyte-like cells (iPS-H) in *immunocompetent* mice by pre-engineering 3D cell co-aggregates with stromal cells (SCs) followed by encapsulation in recently developed biocompatible hydrogel capsules. Notably, upon transplantation, human albumin and α1-antitrypsin (A1AT) in mouse sera secreted by encapsulated iPS-H/SCs aggregates reached a level comparable to the primary Hum-H/SCs control. Further immunohistochemistry of human albumin in retrieved cell aggregates confirmed the survival and function of iPS-H. This proof-of-concept study provides a simple yet robust approach to improve the engraftment of iPS-H, and may be applicable to many stem cell-based therapies.

Liver diseases affect over 600 million people worldwide and result in the death of over 1 million people from chronic and acute liver failure each year^1^. Currently, liver transplantation is the only curative intervention in the treatment of end-stage liver diseases^2^. However, liver transplantation is constrained by the scarcity of donor organs^3^. Cellular therapies designed to treat the increasing number of patients awaiting liver transplantation and proposed as alternative treatments to liver transplantation include hepatocyte transplantation, engineered liver tissues, and bio-artificial liver devices^4^. However, the scarcity of human liver tissue or hepatocytes remains a bottleneck, still hindering the clinical applications of these alternative therapies. Although human hepatocytes (Hum-H) can regenerate *in vivo*, isolated Hum-H rapidly lose their proliferation capacity and differentiated function *ex vivo*^5^. Despite various approaches, limited progress has been made to expand Hum-H *ex vivo*^6^. Alternative sources such as animal hepatocytes are not suitable surrogates due to the safety concerns and the species-specific differences in apolipoprotein expression, cholesterol metabolism, and phase I detoxification enzymes^7^. Therefore, renewable sources of Hum-H are highly desirable to expand cellular therapies for liver failure patients. Moreover, these alternative sources will also facilitate drug screening and liver toxicity studies in drug development.

Human pluripotent stem cells (hPSCs) including embryonic stem cells (ESCs)^8^ and induced pluripotent stem cells (iPSCs)^9^ have unlimited self-renewal potential and can give rise to cells from all three germ layers. They represent promising cell sources to provide sufficient hepatocytes for transplantation^3^, bio-artificial liver devices^4^, liver disease models^10^, targeted gene correction^11^, and drug screening^12^. To generate hepatocytes from hPSCs, various approaches have been pursued over the past decade. Early methods were mainly focused on the differentiation of ESCs^13^,^14^,^15^,^16^,^17^,^18^,^19^,^20^,^21^,^22^ and iPSCs^6^,^22^,^23^,^24^,^25^ into hepatocytes on conventional cell-culture dishes in two-dimensions (2D). Although hepatocyte-like cells were derived with good efficiency, these cells were not fully mature. Their functions were similar to fetal rather than adult Hum-H, as indicated by the expression of α-fetoprotein (AFP) and CYP3A7^13^,^17^,^18^ and lack of key metabolic features^14^. One of the possible explanations might be that 2D culture conditions do not recapitulate the complex three-dimensional (3D) cell-cell and cell-matrix interactions in the liver^26^,^27^. Consequently, the differentiation and maturation of hPSC-derived hepatocyte-like cells in a 3D configuration have been recently proposed based on the simulation of the 3D liver microenvironment and development^28^,^29^,^30^,^31^,^32^,^33^. However, one concern raised with 3D aggregation and differentiation is that the differentiating cells located at different positions in the 3D microenvironment may receive non-uniform hepatic-inducing signals due to differing diffusion time, concentration gradients, and irregular sizes of cell aggregates^28^. To circumvent these limitations, it may be advantageous to combine initial differentiation in 2D with further maturation in 3D. In 2D differentiation, all cells are exposed to uniform differentiating signals from culture medium and surrounding cells, while in 3D cell aggregates the cell-cell and cell-matrix interactions are better recapitulated. To precisely control the size of cell aggregates, a microwell platform has been widely used as a 3D culture system that is capable of producing large numbers of uniform cell aggregates^32^,^34^,^35^.

In most previous 3D culture studies, hPSC-derived hepatocyte-like cells were aggregated alone without non-parenchyma supporting cells (e.g. stromal cells, stellate cells, Kupffer cells, and endothelial cells in liver)^28^,^30^,^31^,^32^,^33^. However, it is well known that heterotypic cell-cell interactions between hepatocytes and their supporting cells are essential for development and maintenance of liver functions^36^,^37^,^38^. Gene expression results demonstrated that co-culturing cell sheets of hPSC-derived hepatocyte-like cells and Swiss 3T3 cells promoted the hepatic maturation of the hepatocyte-like cells^29^. Also, albumin secretion of iPSC-derived hepatocytes was correlated to the configurations of cell-cell contacts^39^. Recently, vascularized and functional human iPSC-derived liver buds were engineered via co-culture with mesenchymal and endothelial cells in immunocompromised mice^26^,^27^. Despite these promising results, we note that previous transplantations were performed in immunocompromised animals^3^,^14^,^17^,^18^,^19^,^23^,^24^,^26^,^27^ to avoid potential immune rejection. Whether and how the hPSC-derived hepatocyte-like cells engraft and function in more clinically relevant immunocompetent models remains largely unexplored. Cell encapsulation and immunoisolation using biocompatible materials and devices allows xenotransplantation in animals with full immune competency^40^. Encapsulation also mitigates the risk of teratoma formation from undifferentiated hPSCs^19^,^41^ in final clinical applications.

In this study, we employed a 3D co-aggregation strategy to improve the function of iPS-H *in vitro* and subsequently a cell encapsulation strategy to achieve the iPS-H engraftment in immunocompetent mice. We first derived iPS-H using a previously published method in a 2D monolayer culture using cytokines in a developmentally appropriate manner^15^,^23^. We then formed 3D cell aggregates of iPS-H together with stromal cells (SCs) using a microwell platform. Importantly, unlike traditional 3D culture where the sizes of cell aggregates were not uniform and not well controlled^42^,^43^, the microwell platform enabled exquisite control on the size of cell aggregates (e.g. ~120 μm of iPS-H/SCs aggregates), mitigating the problems of mass transfer limits and variations in growth factor gradient. The key gene expression, albumin and urea secretion, and cytochrome P450 activity of iPS-H were remarkably improved in cell aggregates of iPS-H/SCs compared to the aggregates of iPS-H alone. After creating sufficient and size-controllable iPS-H/SCs aggregates in microwells, we encapsulated the cell aggregates using recently developed biocompatible alginate capsule formulations and transplanted them into the intraperitoneal cavity of C57BL/6 mice for *in vivo* evaluation. As a control, cell aggregates of primary Hum-H/SCs were prepared, encapsulated, and transplanted in the same manner as iPS-H/SCs. To the best of our knowledge, this is the first *in vivo* iPS-H study using cell encapsulation in immunocompetent animals. Human albumin and α1-antitrypsin (A1AT) secreted from iPS-H was comparable to that from the Hum-H control over 24 days after which the experiment was ended. Gene expression of several hepatic markers (*A1AT*, *albumin*, *MRP3*, CYP2D6, CYP2C9, and *CYP2C19*) was enhanced after transplantation, indicating that iPS-H maintained and even further improved the hepatic functions *in vivo*. Notably, the level of albumin secretion of encapsulated iPS-H in immunocompetent mice was similar to previous reports of unencapsulated cells transplanted in immunocompromised mice^3^,^18^. Moreover, human albumin was positively stained in retrieved iPS-H/SCs aggregates, confirming the engraftment and robust function of iPS-H in the mice.

## Results

**Figure 1:**
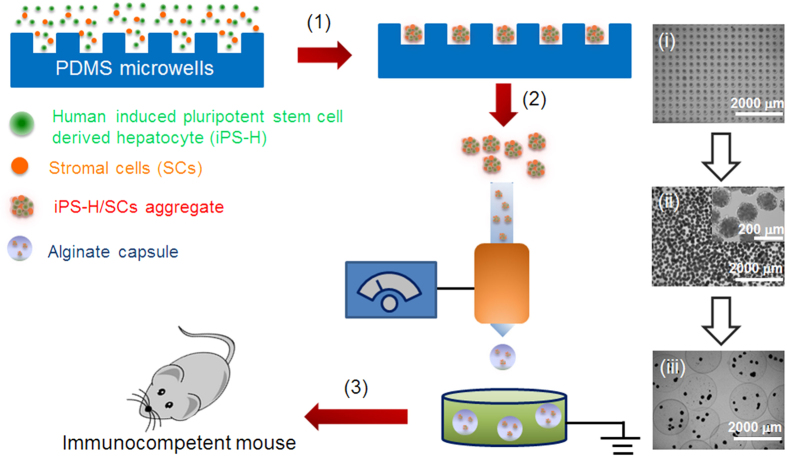
Engineering size-controllable cell aggregates of iPS-H/SCs in PDMS microwells and encapsulation of iPS-H/SCs aggregates in alginate capsules. (1) iPS-H derived from iPSCs and SCs were homogeneously seeded into PDMS microwells and uniform cell aggregates were formed in microwells through overnight gentle shaking. The corresponding image of iPS-H/SCs aggregates in microwells is shown in (i). (2) Cell aggregates were collected from microwells by gentle pipetting and encapsulated in alginate capsules via electrospraying. The corresponding images of iPS-H/SCs aggregates in suspension and capsules are shown in (ii) and (iii). (3) Capsules containing cell aggregates were transplanted into the peritoneal cavity of immunocompetent mouse.

Figure 1 illustrates the experimental procedures and representative images of cell aggregates in microwells, in suspension and in alginate capsules. iPS-H were differentiated from iPSCs on 2D monolayer cultures and then placed into PDMS microwells with SCs. Numerous size-controllable iPS-H/SCs aggregates were formed in 3D microwells. The iPS-H/SCs aggregates were collected from microwells and then encapsulated into alginate capsules using recently developed formulations and a custom-built electrospraying-based cell encapsulator^44^. The capsules containing cell aggregates were transplanted into immunocompetent mice to evaluate the engraftment and function of iPS-H *in vivo*.

### Formation of iPS-H/SCs aggregates in PDMS microwells

We developed a PDMS microwell platform to form size-controllable cell aggregates of various cell types. The diameters of PDMS microwells were from 50 to 800 μm. We started with an INS-1 cell line as a model cell to engineer and optimize cell aggregates in PDMS microwells. After 1 day culture with gentle shaking, cells fell into the microwells, contacted with each other, and formed aggregates (Fig. S1). Importantly, the sizes of cell aggregates were uniformly modulated by the diameters of microwells. Generally, larger cell aggregates were formed in larger size microwells because more cells could be loaded in each well. The microwell platform was scalable to provide sufficient cell aggregates for specific cellular therapy. Given that larger cell aggregates (>200 μm) might cause more inner cell necrosis and difficulty in mass transfer while smaller cell aggregates (<50 μm) might not have enough cell-cell interactions^28^,^45^, we chose microwells of 200 μm diameter to form cell aggregates of ~120 μm in the following experiments. Cell aggregates of primary rat and human hepatocytes (Rat-H and Hum-H) and their mixtures with SCs were easily generated using our microwell platform (Figs S2a, S3a).

**Figure 2:**
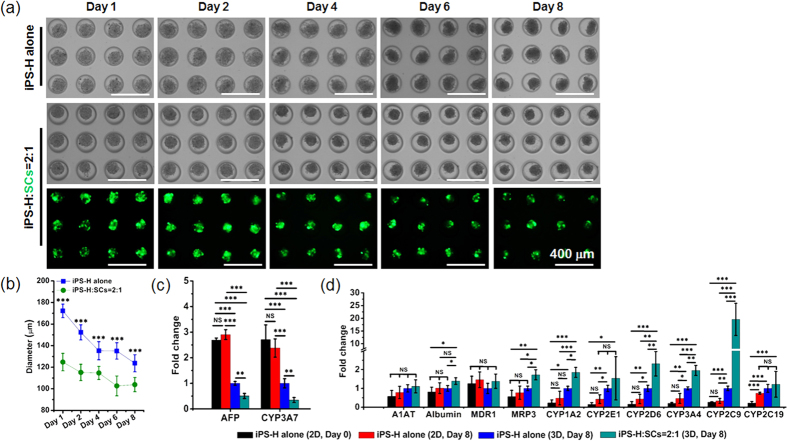
Formation and culture of cell aggregates of iPS-H alone and iPS-H/SCs in PDMS microwells. (**a**) Microscopic images of cell aggregates during 8 days of culture in PDMS microwells. The green color is SCs expressing eGFP proteins. (**b**) The diameter change of cell aggregates during 8 days of culture in PDMS microwells. Mean ± SD (n = 55). ***p < 0.001. The gene expression of hepatocyte markers of iPS-H analyzed with qRT-PCR. (**c**) Fold change of *AFP* and *CYP3A7* gene expression. (**d**) Fold change of characteristic gene expression of protein secretion (*A1AT* and *Albumin*), transporters (*MDR1* and *MRP3*), and cytochrome P450 activity (*CYP1A2*, *CYP2E1*, *CYP2D6*, *CYP3A4*, *CYP2C9*, and *CYP2C19*). Mean ± SD (n = 3). *p < 0.05, **p < 0.01, ***p < 0.001, NS: no significant difference.

Encouraged by the results of cell aggregates of model and primary cells, we pursued cell aggregations of iPS-H alone and mixtures of iPS-H/SCs (iPS-H:SCs = 2:1). The morphology of these cell aggregates at different culture times is shown in Fig. 2a. The cell aggregates were readily formed after 1 day of culture. The size of the cell aggregates was controllable and uniform across the microwells. Interestingly, the sizes of cell aggregates gradually decreased during the initial 4 days culture and then became stable from 4 days to 8 days culture (Fig. 2b). Notably, the addition of SCs affected the size of cell aggregates and resulted in smaller and tighter cell aggregates.

### Gene expression of iPS-H in 2D monolayer and 3D aggregates

The gene expression levels of hepatocyte markers were analyzed using qRT-PCR (Fig. 2c,d). In a preliminary study, we found that co-aggregation with SCs significantly enhanced the hepatic gene expressions of Hum-H (Fig. S3b). Here, we examined how the SCs affected the gene expression of iPS-H over time (Fig. 2c,d). The iPS-H cells were first differentiated from iPSCs in 2D monolayer. iPS-H were then detached and a portion stored for qRT-PCR analysis (iPS-H alone, 2D, Day 0), while others were placed into microwells alone or mixed with SCs to form 3D cell aggregates for 8 days (iPS-H alone, 3D, Day 8 and iPS-H:SCs = 2:1, 3D, Day 8). Concurrently, the remaining iPS-H were continually cultured on 2D monolayer for an additional 8 days (iPS-H alone, 2D, Day 8) as a 2D control. For the genes related to protein secretion, cell aggregates in 3D showed remarkably down-regulated expression of α-fetoprotein (*AFP*) and slightly up-regulated expression of α1-antitrypsin (*A1AT*) and *Albumin* when compared with 2D culture. Compared to cell aggregates of iPS-H alone, the addition of SCs in cell aggregate (i.e. iPS-H/SCs) further reduced *AFP* expression and enhanced *A1AT* and *Albumin* expression. The expression of *CYP3A7* was also reduced in 3D co-aggregates of iPS-H/SCs. The decrease of *AFP* and *CYP3A7* expression in iPS-H/SCs aggregates demonstrated that 3D co-aggregation with SCs significantly enhanced the maturation of iPS-H as these markers are expressed in fetal hepatocytes but not in adult hepatocytes. The slight increase of *A1AT* and *Albumin* expression also verified the higher degree of cell maturation in iPS-H/SCs aggregates. The critical transporter genes, multi-drug resistance 1 (*MDR1*) and multi-drug resistance protein 3 (*MRP3*), were also evaluated in 2D iPS-H and cell aggregates of iPS-H alone and iPS-H/SCs. Although *MDR1* expression did not show obvious difference among the groups, *MRP3* showed significantly higher expression in iPS-H/SCs aggregates than in 2D iPS-H or 3D iPS-H aggregates. Cytochrome P450 genes including *CYP1A2*, *CYP2E1*, *CYP2D6*, *CYP3A4*, *CYP2C9*, and *CYP2C19* (markers of adult human hepatocytes and expressed at significantly lower levels in fetal human hepatocytes) were expressed at higher levels in co-aggregates when compared with aggregates of iPS-H alone.

### Functional assessment of iPS-H *in vitro*

**Figure 3:**
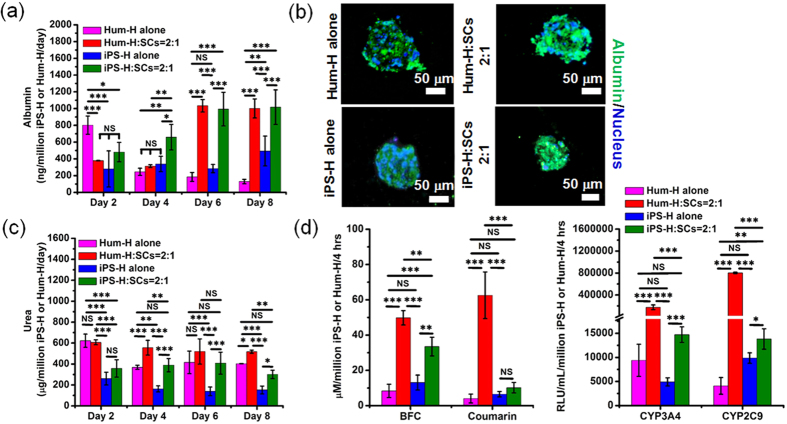
Functional characterization of Hum-H and iPS-H in cell aggregates during 8 days of culture in PDMS microwells. (**a**) Albumin secretion of Hum-H and iPS-H during 8 days of culture. (**b**) Immunostaining images of albumin secreted from Hum-H and iPS-H after 8 days of culture. The green color is albumin and blue color is cell nucleus. (**c**) Urea secretion of Hum-H and iPS-H during 8 days of culture. (**d**) Cytochrome P450 activities of Hum-H and iPS-H after 8 days of culture. Mean ± SD (n = 3). *p < 0.05, **p < 0.01, ***p < 0.001, NS: no significant difference.

The liver-specific functions of iPS-H/SCs aggregates in microwells were evaluated by measuring albumin (Fig. 3a) and urea secretion (Fig. 3c), immunostaining of albumin (Fig. 3b), and cytochrome P450 (CYP) activities (Fig. 3d). As a control, Hum-H alone and Hum-H/SCs aggregates were also prepared and analyzed. Without the support of SCs, albumin secretion from Hum-H aggregates quickly decreased to around 200 ng/million Hum-H/day after 2 days of culture in microwells. Similarly, cell aggregates of iPS-H alone maintained albumin secretion at a lower level during 8 days of culture. However, the addition of SCs significantly improved albumin secretion of Hum-H/SCs and iPS-H/SCs aggregates, which persistently increased during 6 days culture and was stable until 8 days. The amount of albumin secreted from iPS-H/SCs aggregates reached the similar level as Hum-H/SCs aggregates (1,017 ± 205 ng/million iPS-H/day) after 8 days of culture in microwells, which was approximately two times the albumin amount secreted by aggregates of iPS-H alone (493 ± 178 ng/million iPS-H/day). Moreover, as shown in Fig. 3b, albumin was positively stained in cell aggregates of all groups using immunostaining. We found that the effect of SCs on enhanced hepatic function in 3D was not limited to iPS-H and the 2:1 cell ratio as SCs also improved albumin secretion of Rat-H (3:1; Fig. S2b) and Hum-H (1:1; Fig. S3c). Consistent with the effect the SCs had on albumin secretion, the addition of SCs to Rat H (Fig. S2c), Hum-H, and iPS-H (Fig. 3c) also led to a statistically significant increase in urea secretion across the time-course of the experiment. After 6 days culture in microwells, urea secreted from iPS-H/SCs was 408 ± 105 μg/million iPS-H/day, while iPS-H alone only had 139 ± 40 μg/million iPS-H/day. Although the iPS-H/SCs showed slightly decreased urea secretion on Day 8, there was no statistically significant difference when compared to urea secretion on Day 6.

Cytochrome P450 enzymes play key roles in phase I xenobiotic biotransformation. For PSC-derived hepatocyte-like cells, the enzymes are usually expressed at low levels^22^,^23^,^32^ and are rapidly lost after cell manipulation and perturbation. As shown in Fig. 3d, cell aggregates of iPS-H/SCs had increased CYP activity. Specifically, when compared to cell aggregates of iPS-H alone, BFC activity was significantly increased although this nonspecific substrate measures a composite of the activities of several cytochrome P450 enzymes. Coumarin, a specific substrate of CYP2A6, was increased 1.6-fold as compared to iPS-H alone. CYP2C9 and CYP3A4 activities of iPS-H/SCs aggregates were increased 1.4 and 3.0 fold respectively as probed by Luciferin H and CYP3A4-IPA substrates. Overall, these functional evaluations demonstrated that 3D co-aggregation highly enhanced albumin and urea secretion as well as CYP activities of iPS-H. Compared to Hum-H, however, the CYP activities of iPS-H were at a lower level. More work including further optimization of the 3D co-aggregation is still needed to improve the maturation of the iPS-H *in vitro*.

### Encapsulation and transplantation of iPS-H/SCs aggregates

**Figure 4:**
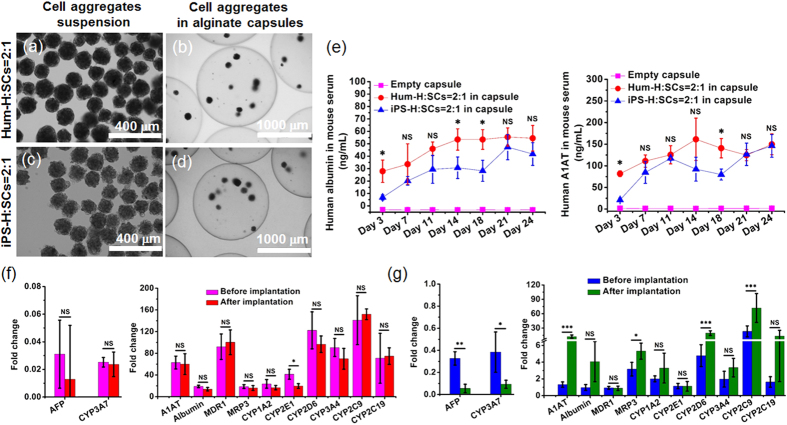
Encapsulation of cell aggregates of Hum-H/SCs and iPS-H/SCs in alginate capsules and functional characterization in immunocompetent mice. (**a,c**) Microscopic images of Hum-H/SCs (**a**) and iPS-H/SCs (**c**) aggregates collected from PDMS microwells. (**b,d**) Microscopic images of Hum-H/SCs (**b**) and iPS-H/SCs (**d**) aggregates in alginate capsules. (**e**) The profile of human albumin and A1AT in mouse serum secreted from encapsulated Hum-H and iPS-H during 24 days of transplantation. (**f,g**) The gene expression of hepatocyte markers of Hum-H (**f**) and iPS-H (**g**) before implantation (Day 2 in microwells) and after implantation (Day 24 in mice) analyzed with qRT-PCR. Mean ± SD (n = 3). *p < 0.05, **p < 0.01, ***p < 0.001, NS: no significant difference.

One of the major challenges working with hPSCs-derived hepatocyte-like cells is to achieve robust hepatic engraftment *in vivo*. Although several reports have shown certain degrees of success in engraftment, these reports to date were all based on studies in immunocompromised animal models^3^,^14^,^17^,^18^,^19^,^23^,^24^,^26^,^27^. To determine the engraftment and function of iPS-H/SCs aggregates in an immunocompetent animal model, we utilized a cell encapsulation and immunoisolation strategy where the encapsulated cells were protected from direct immune cell interactions while still receiving sufficient nutrients and oxygen to survive and function. Prior to encapsulation, the preformed iPS-H/SCs aggregates were collected from microwells after 2 days of culture. Hum-H/SCs aggregates were formed in the same manner and used as a control. Figure 4a,c showed cell aggregates of Hum-H/SCs and iPS-H/SCs in suspension. We noticed that the morphology and roundness of Hum-H/SCs aggregates were slightly different from iPS-H/SCs aggregates. This could be attributed to the inherent differences between cell sources and aggregation ability of Hum-H and iPS-H. We then used an electrospraying technique^44^ to encapsulate the cell aggregates in alginate capsules. The cell aggregates of Hum-H/SCs (Fig. 4b) and iPS-H/SCs (Fig. 4d) were completely encapsulated in the capsules. The diameter of alginate capsules containing Hum-H/SCs and iPS-H/SCs aggregates were approximate 1.5 mm. Each capsule contained approximately 5 ~ 10 cell aggregates. We chose the large size capsule formulations because it was previously found that large size capsules with diameters of ~1–2 mm induced much less foreign body reaction and fibrosis than the smaller size ~500 μm capsules^46^,^47^. We compared the albumin secretion from Hum-H/SCs or iPS-H/SCs cultured in microwells and those encapsulated in capsules. The results indicated that encapsulation and alginate capsules did not seem to affect the albumin secretion or diffusion (Fig. S4).

We implanted the encapsulated cell aggregates into the intraperitoneal cavity of immunocompetent C57BL/6 mice (~4.4 × 10^5^ Hum-H or iPS-H in capsules per mouse). To evaluate the engraftment and function of iPS-H *in vivo* (and indirectly the immune protection of alginate capsules), mouse blood was collected twice a week 3 days post-operation until the experiment was ended on Day 24. The amount of human albumin and α1-antitrypsin (A1AT) in mouse serum was measured via human albumin and A1AT ELISA (Fig. 4e). As early as 3 days post-transplantation, human albumin and A1AT secreted from Hum-H and iPS-H were already detected in mouse serum. In Hum-H/SCs aggregates, the albumin and A1AT secretion gradually increased to 53.5 ng/mL and 161.3 ng/mL, respectively at 14 days and remained at this level for 24 days after transplantation. For iPS-H/SCs, the average level of human albumin and A1AT was slightly lower than the Hum-H control but statistical analysis showed no significant difference after 18 days transplantation. The fact that the iPS-H produced comparable amount of albumin and A1AT to the Hum-H in immunocompetent mice is a promising proof of concept, indicating the successful engraftment and robust function of iPS-H *in vivo*. In addition, when comparing the albumin amount from this study to that in previous reports, we found that albumin secretion level of encapsulated iPS-H in immunocompetent mice was similar to that in immunocompromised mice (i.e. 1.0 × 10^6^ cells injected, 15 days post transplantation^18^ and 2.0 × 10^6^ cells injected, 20 days post transplantation^3^).

**Figure 5:**
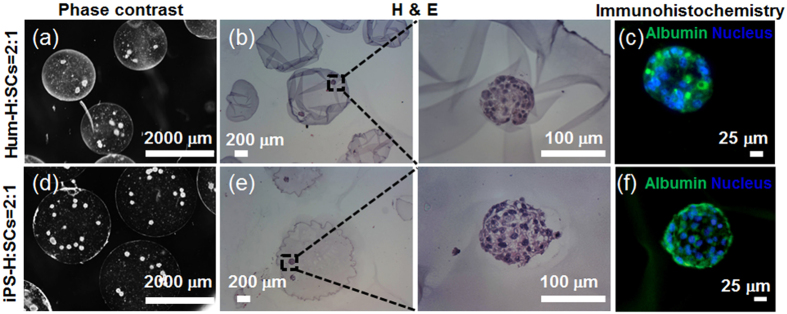
Characterization of retrieved alginate capsules containing Hum-H/SCs and iPS-H/SCs aggregates 18 days after transplantation in immunocompetent mice. (**a,d**) Phase contrast images of Hum-H/SCs (**a**) and iPS-H/SCs (**d**) aggregates in retrieved capsules. (**b,e**) Hematoxylin and Eosin (H&E) staining images of Hum-H/SCs (**b**) and iPS-H/SCs (**e**) aggregates in the capsules. (**c,f**) Immunostaining images of albumin secreted from encapsulated Hum-H (**c**) and iPS-H (**f**). The green color is albumin and the blue color is cell nucleus.

Gene expression of hepatic markers from Hum-H/SCs and iPS-H/SCs aggregates was evaluated after retrieving the capsules (24 days post transplantation) and was compared with that just before transplantation (2 days of culture in microwells). As shown in Fig. 4f, for Hum-H, all the genes except *CYP2E1* showed a comparable expression level before and after transplantation. In contrast, iPS-H had reduced gene expression of *AFP* and *CYP3A7*, and enhanced expression of several other genes including *A1AT*, *Albumin*, *MRP3*, *CYP2D6, CYP2C9, and CYP2C19* after transplantation (Fig. 4g). This data suggested that the alginate capsules protected the cells from direct immune cell attacks and the iPS-H maintained robust and even more mature hepatic functions under *in vivo* environment. In a separate experiment, the alginate capsules were also retrieved, fixed, and processed for imaging and immunostaining (18 days post transplantation). The capsules induced heterogeneous foreign body reactions and fibrosis, exhibiting a wide spectrum of cellular overgrowth around the capsules. Within each mouse, some (~40%) capsules had little to no fibrosis (Fig. 5a,d), while some (~30%) were severely fibrotic (Fig. S5). In general, these human cell-containing capsules had more fibrosis in mice than expected for rodent cell-containing capsules or empty ones using similar formulations^46^,^47^. This might be due to the large species difference between humans and mice^48^. Nevertheless, in the Hum-H/SCs and iPS-H/SCs capsules with minimal fibrosis (Fig. 5a,d), the cell aggregates were intact and had similar cell morphology to those before transplantation. This result could be attributed to the effective immunoisolation of the biocompatible alginate capsules. Representative Hematoxylin and Eosin (H&E) staining of retrieved cell aggregates of Hum-H/SCs and iPS-H/SCs (Fig. 5b,e) showed no evidence of any necrosis in cell aggregates thereby demonstrating sufficient mass transfer and oxygen/nutrient diffusion during the transplantation period. The Hum-H or iPS-H were both stained positive for human albumin proving their survival and function within the encapsulated co-aggregates (Fig. 5c,f). This data combined with the gene expression (Fig. 4f,g) and continuous secretion of human albumin and A1AT in the mouse sera (Fig. 4e) confirmed the successful engraftment and robust function of iPS-H in immunocompetent mice.

## Discussion

Our platform combines the respective advantages of 2D monolayer differentiation and 3D co-aggregation in microwells to enhance the function of iPS-H *in vitro*. Furthermore, we encapsulated iPS-H/SCs aggregates in newly developed alginate capsules and demonstrated *in vivo* engraftment and function of iPS-H in immunocompetent mice. The microwell platform is scalable and tunable allowing control over cell number, aggregate size, cell ratios, and differentiation stages, while the encapsulation in biocompatible materials enables engraftment of functional iPS-H in immunocompetent models.

We first derived hepatocyte-like cells from iPSCs in 2D culture in a step-wise and developmentally appropriate manner via endoderm induction, hepatic specification, hepatoblast expansion, and then hepatic maturation^2^,^15^,^23^. The 2D monolayer culture enables the generation of uniform iPS-H population as the differentiating cells receive homogeneous cytokine and induction signals from the medium^49^. However, these iPS-H are more similar to fetal human hepatocytes^13^,^17^,^18^, which limits their usefulness in *in vitro* platforms and engraftment in animal models such as humanized liver chimeric mice. This is not surprising as liver development is a complex process involving a variety of 3D cell-cell and cell-matrix interactions, which are largely lost in the 2D culture system.

To better recapitulate *in vivo* cell-cell and cell-matrix interactions, we developed a 3D microwell co-aggregation platform to further improve the function of iPS-H. The microwell platform allows precise control over the size of cell aggregates (Fig. S1). The size control can potentially avoid the problems of poor mass transfer in too large aggregates or insufficient cell-cell interactions in too small aggregates, both of which are often seen in non-uniform cell aggregates^28^,^45^,^50^. Therefore, we formed cell aggregates of intermediate size (~120 μm) by controlling the diameters of microwells (Fig. 2a). Interestingly, we found that the iPS-H/SCs co-aggregates seemed to be always smaller than aggregates of the iPS-H alone. Similar phenomena were observed for Rat-H and Hum-H (Figs S2a, S3a), and have also been reported previously for aggregates of rat hepatocytes/stellate cells^51^ and rat islets/hepatocytes^34^. In the iPS-H/SCs co-aggregates, the SCs might behave as a “glue” to tightly connect iPS-H with each other by which cell-cell and cell-matrix interactions were significantly strengthened. The extracellular matrix proteins and cytokines secreted from SCs might have also facilitated the cell contacts and communications which in turn promoted the survival and function of iPS-H^52^.

Compared with iPS-H in 2D or cell aggregates of iPS-H alone, the iPS-H/SCs aggregates generated more functional hepatocytes as indicated by improved gene expression, albumin and urea secretion, and cytochrome P450 activities. At the mRNA level (Fig. 2c,d), *AFP* and *CYP3A7* expression, biomarkers of fetal hepatocytes but not adult hepatocytes, was remarkably down-regulated in iPS-H/SCs aggregates in contrast to consistent expression of *AFP* and *CYP3A7* in 2D cultures^13^,^17^,^18^. Moreover, the iPS-H/SCs showed up-regulated gene expression of *A1AT* and *Albumin*. The critical transporter gene, *MRP3*, was also significantly up-regulated in iPS-H/SCs aggregates. More importantly, the genes related to metabolic cytochrome P450 functions of hepatocytes were up-regulated when SCs were incorporated in the iPS-H aggregates. Given that the genes of *CYP1A2*, *CYP2D6*, *CYP3A4*, *CYP2C9*, and *CYP2C19* are more robustly expressed in adult hepatocytes^2^, co-aggregation with SCs in 3D microwells significantly stabilized and enhanced metabolic function of iPS-H. At the protein level (Fig. 3a,b), SCs in cell aggregates markedly improved albumin secretion from iPS-H. Albumin in cell aggregates of iPS-H alone was consistently secreted at a lower level. In contrast, the amount of secreted albumin gradually increased as the culture time prolonged in iPS-H/SCs aggregates. The albumin amount at day 8 (~1.1 μg/million iPS-H/day) was similar to previous 3D aggregation experiments^33^,^39^ and seemed better than several studies using 2D culture^25^, 3D dynamic perfusion^31^, and 3D co-culture with endothelial cells^53^. Consistent with albumin, urea secretion was also elevated in iPS-H/SCs aggregates, which was approximately two times that in cell aggregates of iPS-H alone (Fig. 3c). The urea production of iPS-H in co-aggregates at day 6 (~400 μg/million iPS-H/day) appeared better than previous 3D cultures of hESCs-derived hepatocytes without supporting cells^28^,^54^. At the cytochrome P450 activity level (Fig. 3d), CYP3A4, CYP2A6, and CYP2C9 activities of iPS-H/SCs aggregates increased approximately 1.4 ~ 3.0 fold when compared to cell aggregates of iPS-H alone. It is well known that cytochrome P450 enzymes are of great importance in phase I xenobiotic biotransformation. Our results suggest that SCs in cell aggregates enhanced metabolic function of iPS-H. Taken together, these data suggest that the incorporation of SCs in cell aggregates is definitely beneficial to the enhanced function of iPS-H. While the exact mechanism of SCs beneficial effect requires further investigation, it is speculated that the SCs facilitated physiologically relevant cell-cell and cell-matrix supports for iPS-H in 3D aggregates. The importance of these cell-cell and cell-matrix interactions has been well documented in the maintenance and function of primary hepatocytes^36^,^55^,^56^,^57^. The physical contacts, deposited extracellular matrix proteins, and paracrine signaling from SCs could also play important roles in the formation of 3D cell aggregates^27^ as well as the enhanced function of iPS-H^29^.

Takebe *et al.* recently developed an elegant protocol to engineer iPSC-derived liver buds by co-culturing iPSCs-derived endodermal cells with mesenchymal and endothelial cells^26^,^27^. However, the “liver buds” were not demonstrated to have enhanced hepatocyte phenotype or function, and similar to prior studies their experiments were performed in immunocompromised animals^3^,^14^,^17^,^18^,^19^,^23^,^24^,^26^,^27^. Despite great efforts from many groups, there are few reports of robust iPS-H engraftment, repopulation, and function *in vivo*. Although the reasons underlying these difficulties remain unclear, species differences between iPS-H and the mouse recipient may be preventing both iPS-H engraftment and repopulation. As a consequence we strove to use cell encapsulation to bypass this problem altogether and directly implant robust and functional iPS-H in preformed engineered aggregates and capsules. This approach not only avoids direct immune cell rejection and potentially circumvents the engraftment problem but also mitigates the safety concerns due to PSC-derived teratomas^19^,^41^. It is notable that despite preventing direct immune cell interactions, cell encapsulation did not seem to completely eliminate the immune responses induced by foreign cells. We measured the immune rejection-related cytokines Interleukin 2 (IL-2) and Interferon gamma (IFN-γ) in the sera of mice implanted with empty capsules and encapsulated cells (Fig. S6). While IFN-γ remained similar levels for all mice, IL-2 was elevated in mice with encapsulated cells. However, the newly developed alginate capsule formulations^46^ still maintained the survival and function of iPS-H in immunocompetent mice. Although a fraction of the implanted capsules were fibrotic (probably due to the large species difference between human cells and mouse recipients), the majority of them had minimal fibrosis and contained functional cell aggregates 24 days after transplantation. Interestingly, it appeared that capsules containing iPS-H/SCs induced a higher level of fibrosis than those encapsulating Hum-H/SCs (Fig. S5), suggesting the cytokines secreted from iPS-H and Hum-H might be different and those from iPS-H might elicit greater host responses. Future work will be performed to analyze and compare the types and amounts of cytokines derived from Hum-H and iPS-H. Nevertheless, the beneficial effect of cell encapsulation was obvious as the hepatic gene expression of immunoisolated iPS-H was stable and several of the critical genes were even elevated during transplantation in immunocompetent mice. The *in vivo* environment might facilitate the maturation of iPS-H; similar maturation effect of *in vivo* environment has been demonstrated in hPSCs-derived pancreatic cells^41^. More importantly, human albumin and A1AT secreted from iPS-H/SCs aggregates was detected in mouse sera for 24 days (Fig. 4e). The level of secretion was generally comparable to Hum-H/SCs aggregates and previous reports using immunocompromised mice^3^,^18^.

While these data are promising, further work is needed to develop iPS-H for clinical uses. For example, it would be valuable to evaluate the function of encapsulated iPS-H/SCs aggregates in a liver drug failure model (i.e. CCl_4_ treated animals^24^) or in a genetic model (i.e. with fumarylacetoacetate hydrolase (FAH) gene^58^). Furthermore, other types of supporting cells will likely be needed to further enhance the maturation of iPS-H. To achieve long-term or permanent function and engraftment, new encapsulation systems will also be required that can either completely escape the foreign body reaction or become functionally vascularized. Lastly, the method of combining co-aggregation in microwells and cell encapsulation using biocompatible formulations may be applicable for many cell therapies such as stem cell-derived pancreatic cells^59^ for type 1 diabetes.

## Methods

### Fabrication of PDMS microwells

PDMS microwells were fabricated using standard soft lithography at Cornell NanoScale Facility (CNF). Briefly, the photomask with circular micropatterns was prepared using a mask writer (DWL2000, Heidelberg Instruments). The silicon wafer was spin-coated with SU-8 2150 photoresist (MicroChem) at 500 rpm for 40 sec and then 2500 rpm for 30 sec. The wafer was covered with the photomask and exposed with a UV photolithography machine (ABM Contact Aligner) for 32 sec. After being developed and post-baked, the SU-8 master wafer was fabricated. The master wafer has circular microposts with various diameters and thickness of 220 μm. The master wafer was then used to create PDMS (Sylgard 184, Dow Corning) microwells. A mixture (10:1, w-w) of Sylgard 184 silicone elastomer components was casted onto the master wafer, cured at 60 °C overnight, and peeled off from master to obtain PDMS microwells.

### Culture of iPS-H, stromal cells, and primary human hepatocyte

LN4 (a subclone of iPS.C2a with higher uniformity in growth and differentiation) were used for all experiments. iPS.C2a originated from the laboratory of Dr. Stephen Duncan at the Medical College of Wisconsin and was initially reprogrammed from human foreskin fibroblasts^23^. Undifferentiated iPSCs were maintained and differentiated into iPS-H as previously described^23^. In brief, iPSCs were cultured in monolayer on Matrigel (Becton Dickinson), and directed differentiation was achieved by sequential exposure to activin A, bone morphogenic protein 4 (BMP4) and basic fibroblast growth factor (bFGF), hepatocyte growth factor (HGF), and oncostatin M (OSM).

J2-3T3 stromal cells were obtained from Dr. Howard Green at Harvard Medical School. mCherry and eGFP expressing J2-3T3 were generated using lentiviral vectors expressing mCherry (Addgene) and eGFP (Addgene), respectively. They were then sorted for high expressing fluorescent cells and grown in DMEM (Invitrogen) with 10% (v/v) fetal bovine serum (FBS, Invitrogen), 100 units/mL penicillin (Invitrogen), and 100 μg/mL streptomycin (Invitrogen). J2-3T3 cells were grown on a tissue culture plate and irradiated with 4000 rad using a Cesium source irradiator prior to use in all experiments.

Primary human hepatocytes were obtained through the Liver Tissue Cell Distribution System which was funded by NIH Contract # HHSN276201200017C.

### Formation of cell aggregates of iPS-H alone and iPS-H/SCs in PDMS microwells

PDMS microwells were autoclaved, placed in a 12-well plate, and coated with 1% (w/v) Pluronic^®^ F127 (Sigma) solution before cell seeding to prevent cell attachment on PDMS surface and facilitate formation of cell aggregates.

To form cell aggregates, cell suspensions of iPS-H alone (2.0 × 10^6 ^cells) and iPS-H/SCs mixture (iPS-H:SCs = 2:1, total 2.0 × 10^6^ cells) were added to each well of 12-well plate with PDMS microwells inside. After 4 hours of static culture, the cells that were adhered to the interspace between microwells were removed by medium change. The cells that fell into the microwells formed cell aggregates after overnight culture with gentle shaking. The cell aggregates were cultured in microwells for 8 days. The differentiation medium (RPMI-1640 with 2% (v/v) B27 with insulin, 1% (v/v) nonessential amino acids, 100 units/mL penicillin, and 100 μg/mL streptomycin (all from Invitrogen) and 20 ng/mL HGF (R&D Systems)) was changed every 2 days. The cell aggregates of Hum-H alone and Hum-H/SCs were prepared in the same way as the case of iPS-H.

### Quantitative real time reverse transcription polymerase chain reaction (qRT-PCR)

The gene expression of iPS-H alone and iPS-H/SCs in 2D and 3D microwell was measured using qRT-PCR. After iPS-H was differentiated from iPSCs on 2D culture (iPS-H alone, 2D, Day 0), a portion of them was detached and placed into microwells alone or mixed with SCs to culture 3D cell aggregates for 8 days using aforementioned method. Parallel to the 3D microwell culture, a portion of undetached iPS-H was continually cultured on 2D for an additional 8 days (iPS-H alone, 2D, Day 8). After 8 days of culture, cell aggregates of iPS-H alone (iPS-H alone, 3D, Day 8) and iPS-H/SCs (iPS-H:SCs = 2:1, 3D, Day 8) in microwells were washed with PBS for 3 times and then collected into Eppendorf tubes. After centrifuge at 1000 rpm for 5 min, the supernatant PBS was removed and the pellets of cell aggregates were flash frozen in liquid nitrogen. The preparation of cell pellets of Hum-H alone and Hum-H/SCs aggregates (Day 8 in microwells) was same as the case of iPS-H.

Cells were then lysed with RLT buffer and total RNA was isolated using an RNAeasy Kit (Qiagen). Isolated RNA was then treated with DNase (NEB) and RNA cleaned up with the RNA Clean and Concentrator kit (Zymo Research). 2 μg of RNA was used for cDNA synthesis (iScript cDNA synthesis kit, Biorad). qRT-PCR was performed using SYBR Green PCR (Biorad) on a Biorad CFX PCR machine. The gene expression data was normalized to the group of iPS-H alone, 3D, Day 8 using β-actin as a housekeeper gene. Human-specific primers were used and the sequences of primers are shown in the supplementary information.

### Albumin and urea secretion

Albumin and urea secretion were analyzed by measuring the concentration of albumin and urea in culture medium. The medium was collected and replaced with fresh medium every 2 days. The collected medium was centrifuged at 1000 rpm for 5 min. The supernatant was stored at −20 °C for analysis of albumin and urea secretion. Secreted albumin in the supernatant was quantified by an enzyme-linked immunosorbent assay (ELISA) kit using sheep anti-human albumin antibodies (Bethyl Labs) and horseradish peroxidase detection (3,3′,5,5′-tetramethylbenzidine, Invitrogen). Urea concentration was assayed using a colorimetric endpoint assay using diacetylmonoxime with acid and heat (Stanbio Labs). The albumin secretion from cell aggregates in microwells and in alginate capsules was also compared *in vitro*. After 2 days of culture in microwells, a portion of cell aggregates was collected from microwells and encapsulated in alginate capsules. Cell aggregates in microwells and capsules were then cultured for another 2 days in parallel. The secreted albumin in medium were analyzed using aforementioned method.

### Cytochrome P450 activity

After 8 days of culture, cell aggregates of iPS-H alone and iPS-H/SCs in PDMS microwells were analyzed for cytochrome P450 activity. The substrate medium was high glucose DMEM without phenol red (Gibco) supplemented with 100 units/mL penicillin (Gibco) and 100 μg/mL streptomycin (Gibco). Four substrates divided into two groups were added into medium at proper dilution ratios: group 1 was composed of CYP3A4-IPA for CYP3A4 (dilution: 1:1000, Promega) and 7-benzyloxy-trifluoromethylcoumarin (BFC) (50 μm, Sigma) for multiple CYP450 isoforms; group 2 was composed of 6′deoxyluciferin (Luciferin H) (dilution: 1:50, Promega) for CYP2C9 and Coumarin (50 μm, Sigma) for CYP2A6. The spent medium was removed and cell aggregates were washed with PBS for 3 times. The cell aggregates were incubated with group 1 substrate medium for 4 hours at 37 °C. After 4 hours, the medium was placed in Eppendorf tubes and frozen at −20 °C for further analysis. The cell aggregates were washed with PBS 3 times and then incubated with group 2 substrate medium for another 4 hours at 37 °C. After 4 hours, the medium was placed in Eppendorf tubes and frozen at −20 °C for further analysis. Metabolite conjugates formed from BFC and coumarin were incubated with β-glucuronidase/arylsulfatase (Roche) for 2 hour at 37 °C. Samples were diluted 1:1 in quenching solution and formation of metabolites was quantified with a fluorescence microplate reader (Molecular Devices) as described elsewhere^60^. Metabolite conjugates formed from Luciferin H and CYP3A4-IPA were processed and analyzed per Promega protocol and analyzed using a microplate luminometer (Molecular Devices). CYP450 activity of Hum-H alone and Hum/SCs aggregates was also measured using aforementioned method.

### Immunocytochemistry

After 8 days of culture, Hum-H/SCs and iPS-H/SCs aggregates in PDMS microwells were washed with PBS 3 times and fixed with 10% formalin for 30 min. After 3 washes with PBS, the cell aggregates were collected from the PDMS microwells and embedded into HistoGel^TM^ (Thermo Scientific) on ice. Histogel pellets were processed, embedded, and sectioned for immunocytochemistry. Sections were stained with a primary antibody against human albumin (1:500, Sigma) followed by secondary Alexa 488-conjugated donkey-anti-mouse antibody (Invitrogen). Control slides were only stained with secondary conjugated antibody. Images were obtained using a Nikon Eclipse TE200 microscope.

### Encapsulation of iPS-H/SCs and Hum-H/SCs aggregates in alginate capsules

The alginate capsules with cell aggregates of iPS-H/SCs and Hum-H/SCs were made with a custom-built electrospraying-based cell encapsulator^44^. After 2 days of culture in microwells, cell aggregates were mixed with 1.6% (w/v) sterile alginate (Pronova SLG20, NovaMatrix, dissolved in 0.8% (w/v) sodium chloride) and gently pipetted several times. The suspension of cell aggregates was loaded in a 5 mL syringe with 16G blunt needle mounted on a vertical syringe pump (Harvard Apparatus). The suspension was then sprayed into a crosslinking bath with 100 mM calcium chloride and 5 mM barium chloride solution at an electrical strength of approximately 5.8 kV. The crosslinking bath was located 2 cm beneath the syringe needle. The formulation and process parameters described above resulted in capsules with diameters between 1 to 2 mm which were recently found to be less fibrotic than those with smaller, 500 μm diameter^47^. After 2 washes with sterile PBS, the alginate capsules containing cell aggregates were transplanted into C57BL/6 mice (The Jackson Laboratory).

### Transplantation, blood sample collection, and retrieval

All procedures were approved by and performed according to the guidelines of the Institutional Animal Care and Use Committee at the Cornell University. The immunocompetent C57BL/6 mice were anesthetized using 3% (v/v) isofluorane in oxygen and maintained at the same rate throughout the procedure. The mouse abdomen was shaved and alternately scrubbed with betadine and isopropyl alcohol to create a sterile field. A ~3 mm incision was made along the midline of the abdomen and the peritoneum was exposed using blunt dissection. The peritoneum was then grasped with forceps and a ~3 mm incision was made along the linea alba. The alginate capsules containing cell aggregates (~4.4 × 10^5^ Hum-H or iPS-H in capsules for 1 mouse) were injected into the intraperitoneal cavity through the syringe. Empty capsules without cells were injected as a control group. The incision was sutured using 5–0 taper tipped polydioxanone absorbable sutures (PDS II) and the skin was closed using a wound-clip. Starting 3 days post-transplantation, approximately 100 μL blood was collected from the orbital sinus of each mouse, twice a week. The blood was placed into microtainer tubes (Becton Dickinson) containing blood/serum separation gel. Human albumin and A1AT in mouse serum were measured using the human albumin and A1AT ELISA kit (Bethyl Labs). Mouse IFN-γ and IL-2 in mouse serum were assayed using the mouse IFN-γ and IL-2 ELISA kits (eBioscience). After 18 days transplantation, the mice were sacrificed and the alginate capsules containing encapsulated Hum-H and iPS-H were retrieved and fixed in 10% formalin. Immunohistochemistry of human albumin was performed in a similar way as previously described. 24 days after transplantation, the retrieved alginate capsules containing cell aggregates were dissolved in 50 mM ethylenediaminetetraacetic acid (EDTA) and 10 mM 4-(2-hydroxyethyl)-1-piperazineethanesulfonic acid (HEPES) solution to harvest the Hum-H and iPS-H cells. The obtained cells were centrifuged, washed with PBS, and flash frozen in liquid nitrogen. The gene expression before transplantation (Day 2 in microwells) and after transplantation (Day 24 in mice) was analyzed and compared using aforementioned qRT-PCR.

### Statistical analysis

All data were expressed as mean ± standard deviation (SD). SD was illustrated by error bars in Figures. All experiments were performed for at least 3 times from independent differentiations. For each experiments at least 3 replicates were included, and the mean and SD were calculated based on these replicates. Statistical analysis was performed using two-way analysis of variance (ANOVA). *P* values less than 0.05 (*p* < 0.05) were considered to be statistically significant difference.

## Additional Information

**How to cite this article**: Song, W. *et al.* Engraftment of human induced pluripotent stem cell-derived hepatocytes in immunocompetent mice via 3D co-aggregation and encapsulation. *Sci. Rep.* **5**, 16884; doi: 10.1038/srep16884 (2015).

## Supplementary Material

Supplementary Information
