# Supplementary material for: Engraftment of human induced pluripotent stem cell-derived hepatocytes in immunocompetent mice via 3D co-aggregation and encapsulation
Source: Sci Rep. 2015 Nov 23;5:16884. doi: 10.1038/srep16884 (PMC4655358; doi:10.1038/srep16884)
Supplement: Supplementary Information [file srep16884-s1.pdf]

Engraftment of human induced pluripotent stem cell-derived hepatocytes in immunocompetent mice via 3D co-aggregation and encapsulation

Wei Song[1], Yen-Chun Lu[1], Angela S. Frankel[2], Duo An[1], Robert E. Schwartz[2, *], Minglin Ma[1, *]

[1]Department of Biological and Environmental Engineering, Cornell University, Ithaca, NY 14850, USA

[2]Division of Gastroenterology & Hepatology, Department of Medicine, Weill Medical College of Cornell University, New York, NY 10021, USA.

Materials and Methods

Formation of INS-1 832/13 cell aggregates

INS-1 832/13 cells were cultured in RPMI 1640 medium (Gibco) supplemented with 2 mM glutamine (Gibco), 1 mM sodium pyruvate (Gibco), 10% (v/v) fetal bovine serum (FBS, Gibco), 10 mM 4-(2-hydroxyethyl)-1-piperazineethanesulfonic acid (HEPES, Sigma), 100 units/mL penicillin (Gibco), 100 μg/mL streptomycin (Gibco),

250 ng/mL amphotericin B (Gibco), and 50 μM β-mercaptoethanol (Gibco). Cells were plated at a density of ~10,000 cells/cm^2 and grown at 37 °C and 5% CO_2 incubator to ~80% confluence over ~5 days. The medium was changed every 3 days.

To form cell aggregates, INS-1 cells were detached from cell culture flask using 0.05% trypsin/EDTA (Life Technologies). The number of dissociated INS-1 cells was counted and the cell concentration was adjusted to 2.0×10^6 cells/mL. 1 mL of cell suspension was added to each well of 12-well plate with PDMS microwells inside. After 4 h static culture, the cells adhered on the interspace between microwells were removed by medium change. After overnight culture with gentle shaking, the cells fell into the PDMS microwells and formed aggregates. The medium was changed every 3 days.

Formation of rat hepatocytes/stromal cells (Rat-H/SCs) aggregates

Primary rat hepatocytes (Rat-H) were isolated and purified by a modified procedure of Seglen [1] and maintained in high glucose DMEM (Sigma) supplemented with 10% (v/v) FBS (Gibco), 0.5 U/mL insulin (Lilly), 7 ng/ml glucagons (Bedford Laboratories), 7.5 μg/ml hydrocortisone (Sigma), and 1% (v/v) penicillin-streptomycin (Invitrogen).

To form cell aggregates, cell suspension (2.0×10^6 cells) of Rat-H alone and a 3:1

mixture of Rat-H and SCs was added to each well of 12-well plate with PDMS microwells inside. After 4 h static culture, the cells adhered on the interspace between microwells were removed by medium change. After overnight culture with gentle shaking, the cells fell into the PDMS microwells formed aggregates. The medium was changed every 2 days.

The sequence of primers

Gene Name	Forward 5'-3'	Reverse 3'-5'
A1AT	ACGAGACAGAAGACGGCATT	CCCTCTGGATCCACTGCTT
AFP	CCTACAATTCTTCTTTGGGCT	AGTAACAGTTATGGCTTGGA
Albumin	GGAATGCTGCCATGGAGATCTGC	CCTTCAGTTTACTGGAGATCG
MDR1	CTAATGCCGAACACATTGGA	CAGTCGCTTTATTTCTTTGCC
MRP3	GGAGGGCATCAGGCAGGGTGA	GACACAAAGGCCTTCTCGGCGT
CYP1A2	ATGGCATTGTCCCAGTCTGTT	TGGCTCTGGTGGACTTTTCAG
CYP2E1	CTGACCACCCTCCGGAACTA	ATGTAGGCTATGACGTTGCA
CYP2D6	ACCTAGCTCAGGAGGGACTG	GCTGGGATATGCAGGAGGAC
CYP3A4	AGTCGCCTCGAAGATACACA	GGACAGAGCTTTGTGGGACT
CYP3A7	TGCTCTAGTCAGAGTCCTTCAGA	CAGGCTCCACTTACGGTCTCA

CYP2C9	GGACATGAACAACCCTCAGGA	TCAACTGCAGTGTTTTCCAAGC
CYP2C19	TGGACATCAACAACCCTCGG	AGTCAGCTGCAGTGATTACCA

Calculation of iPS-H amount in cell aggregates for transplantation

Surface area of one well of 12-well plate (Corning): $S_{plate} = 3.8\ \text{cm}^2$

Number of microwells in a PDMS mold: $N_{well} = 1000$

Radius of one microwell: $R_{well} = 0.01\ \text{cm}$

Surface area of one microwell: $S_{well} = \pi R_{well}^2 = \pi\,(0.01)^2 = 3.14\times10^{-4}\ \text{cm}^2$

Total surface area of microwells in a PDMS mold:

$$S_{total} = S_{well} \times N_{well} = 3.14\times10^{-4} \times 1000 = 0.314\ \text{cm}^2$$

The fraction of iPS-H in cell aggregate of iPS-H/SCs: $r = 2/(2+1) = 2/3$

Number of total cells seeded in one well of 12-well plate: $N_{total} = 2\times10^6$ cells

Number of iPS-H in a PDMS mold:

$$N_{iPS\text{-}H} = (N_{total} / S_{plate}) \times S_{total} \times r = (2\times10^6 / 3.8) \times 0.314 \times 2/3 = 1.1\times10^5 \text{ cells}$$

Because cell aggregates of iPS-H/SCs collected from 4 PDMS microwell molds were encapsulated in capsules for transplantation into 1 mouse, the number of transplanted iPS-H: $N_{iPS\text{-}H} \times 4 = 4.4\times10^5$ cells

Figures

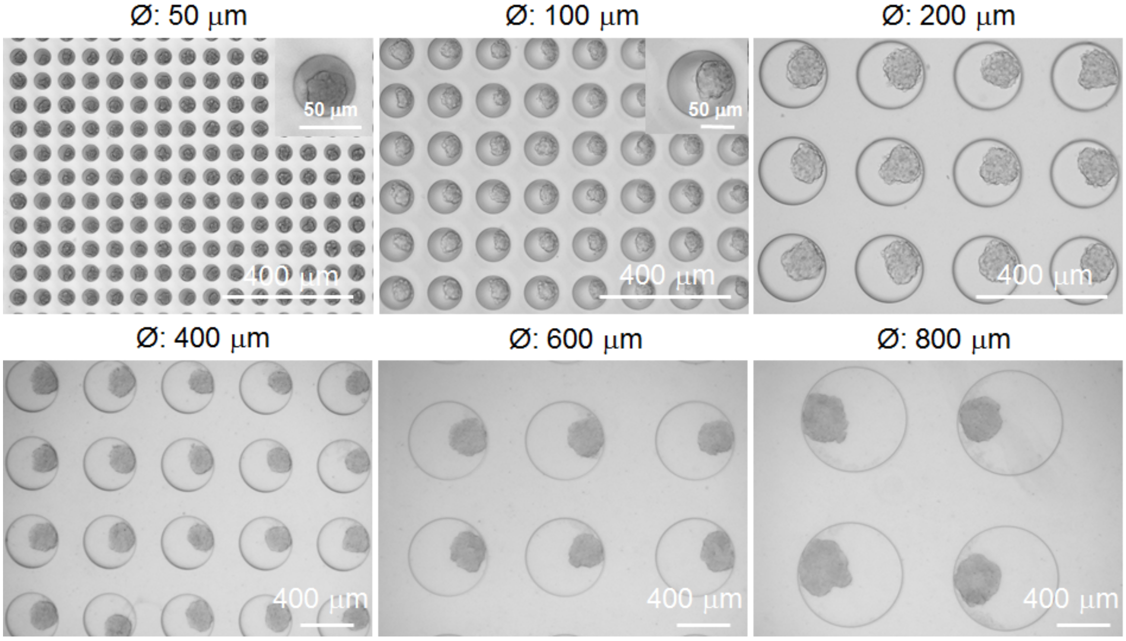

Figure S1. Different sizes of INS-1 cell aggregates formed in PDMS microwells of varied diameters. The diameters of PDMS microwells are from 50 to 800 μm. The inserts are magnified images of one microwell to clearly show cell aggregates inside.

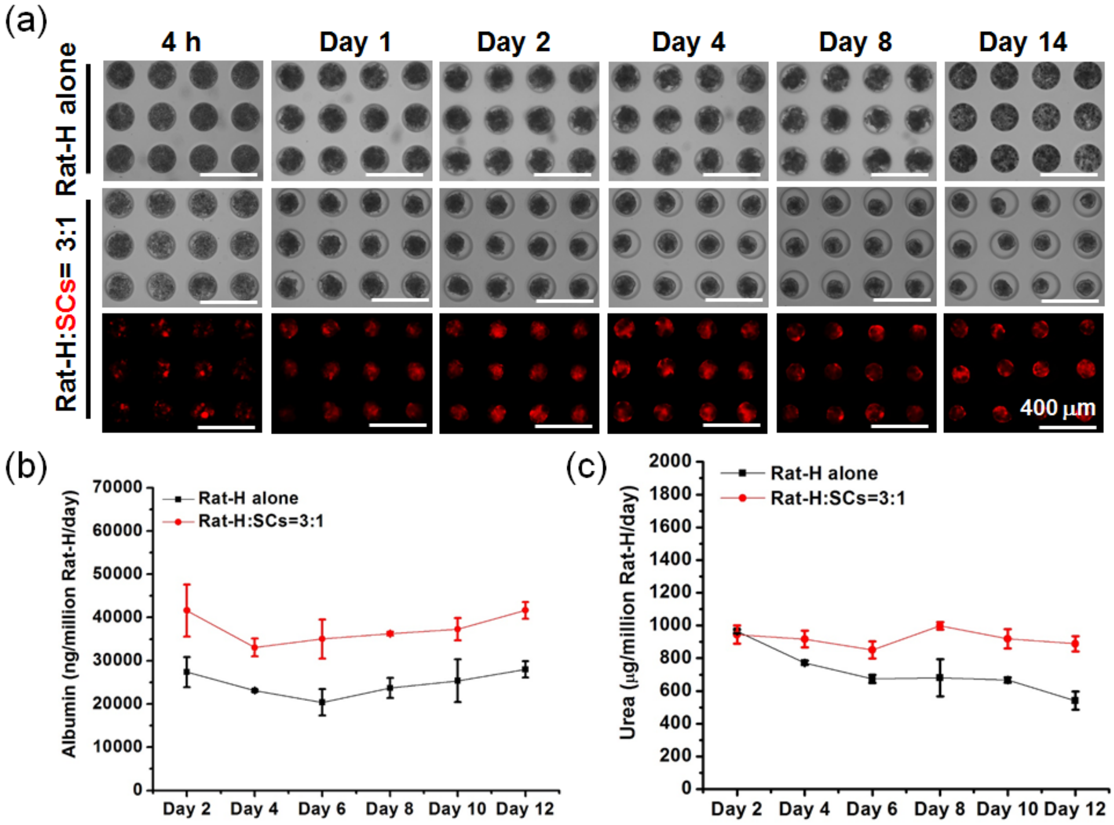

Figure S2. Morphology and functional characterization of Rat-H and Rat-H/SCs aggregates cultured in PDMS microwells. (a) Microscopic images of morphology change of Rat-H and Rat-H/SCs aggregates during 14 days of culture in PDMS microwells. The red color is SCs expressing mCherry proteins. (b) Albumin secretion of Rat-H in cell aggregates of Rat-H alone and Rat-H/SCs. (c) Urea secretion of Rat-H in cell aggregates of Rat-H alone and Rat-H/SCs. Mean ± SD (n = 3).

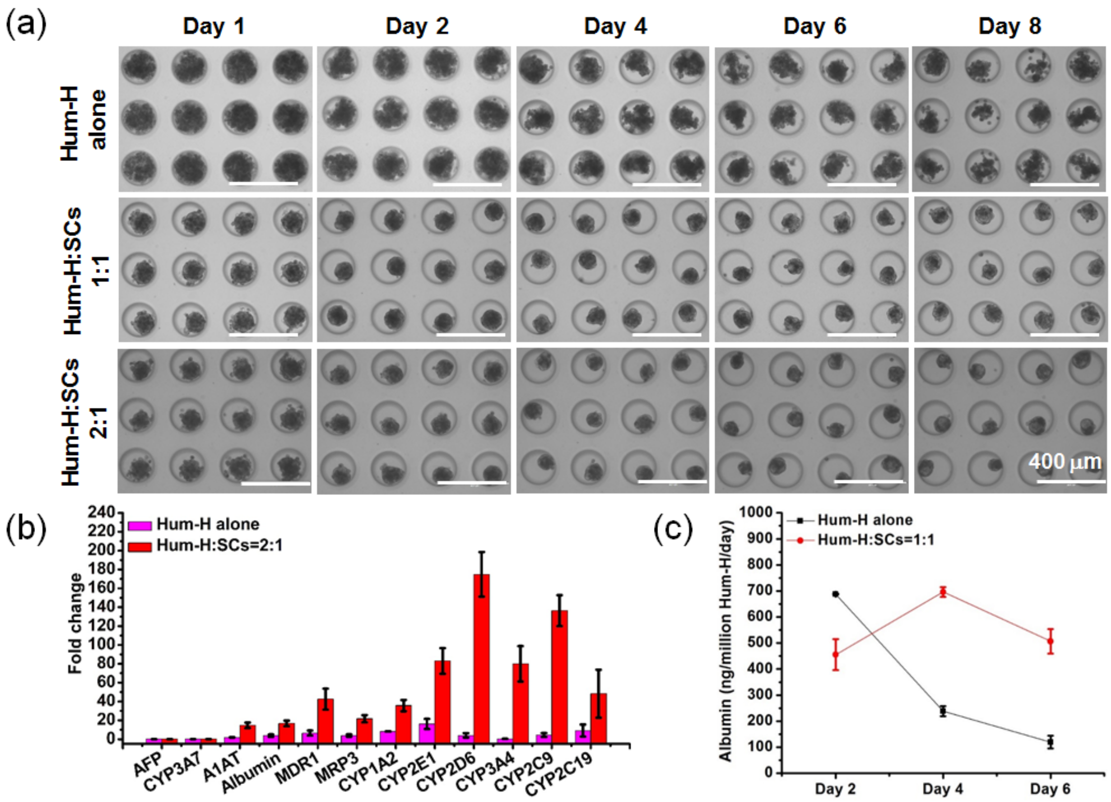

Fig. S3. Morphology and functional characterization of Hum-H and Hum-H/SCs aggregates cultured in PDMS microwells. (a) Microscopic images of morphology change of Hum-H and Hum-H/SCs aggregates during 8 days of culture in PDMS microwells. (b) The gene expression of hepatocyte markers of Hum-H in aggregates of Hum-H alone and Hum-H/SCs after 8 days of culture in PDMS microwells. (c) Albumin secretion of Hum-H in cell aggregates of Hum-H alone and Hum-H/SCs. Mean ± SD (n = 3).

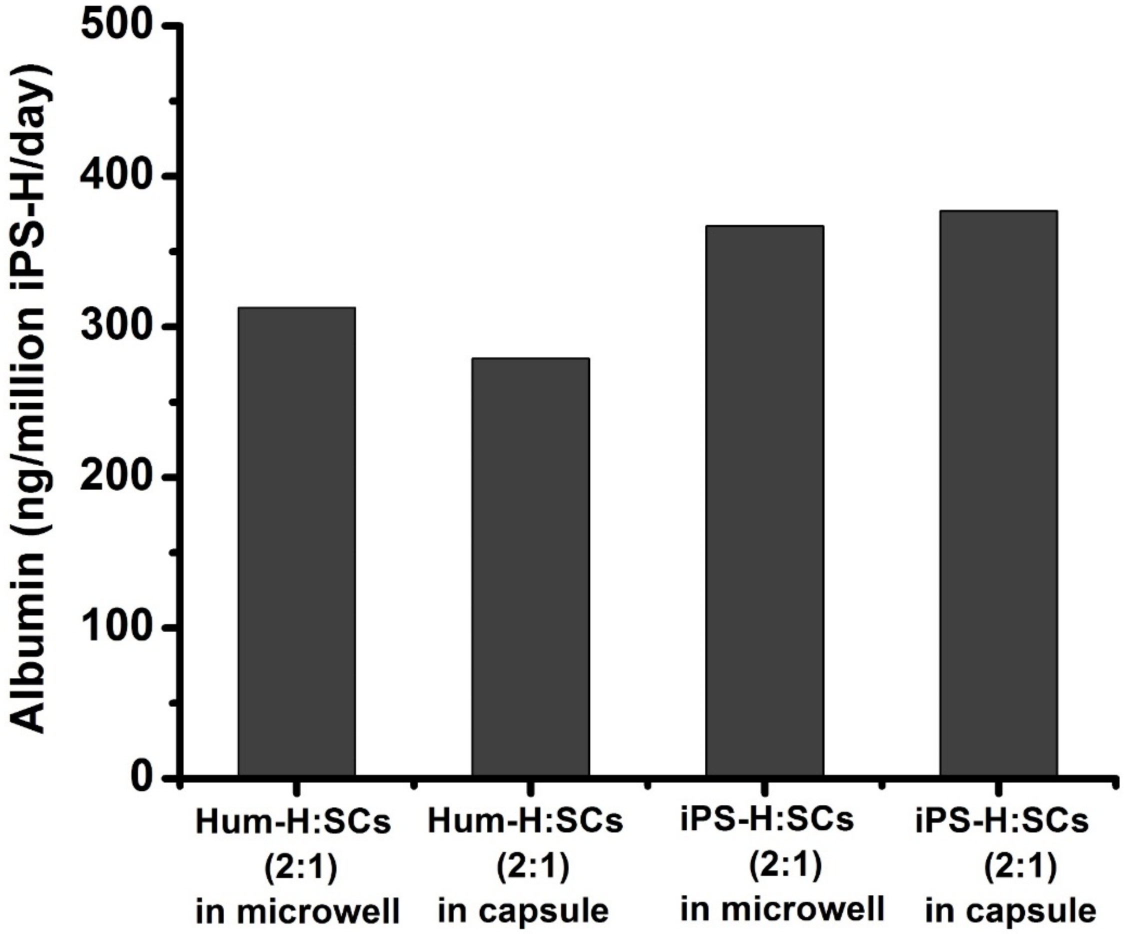

Fig. S4. The comparison of albumin secretion from Hum-H and iPS-H between in microwells and in capsules.

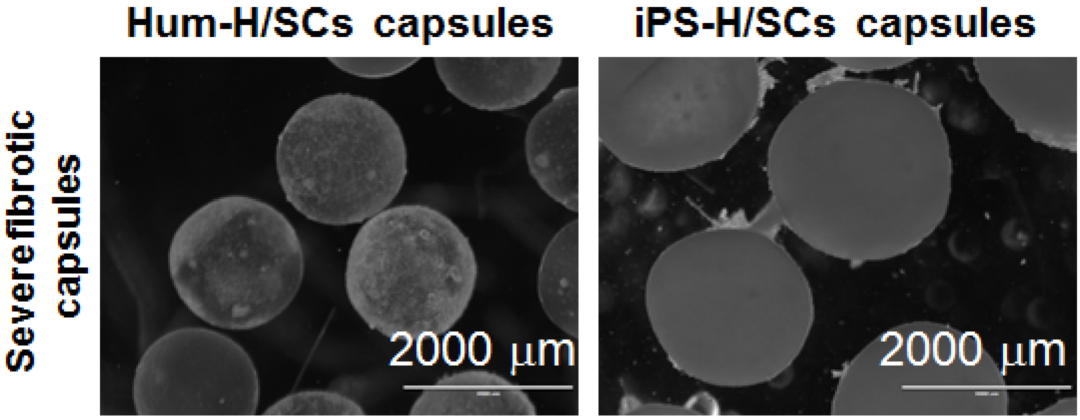

Fig. S5. Microscopic images of severely fibrotic, retrieved alginate capsules containing Hum-H/SCs and iPS-H/SCs.

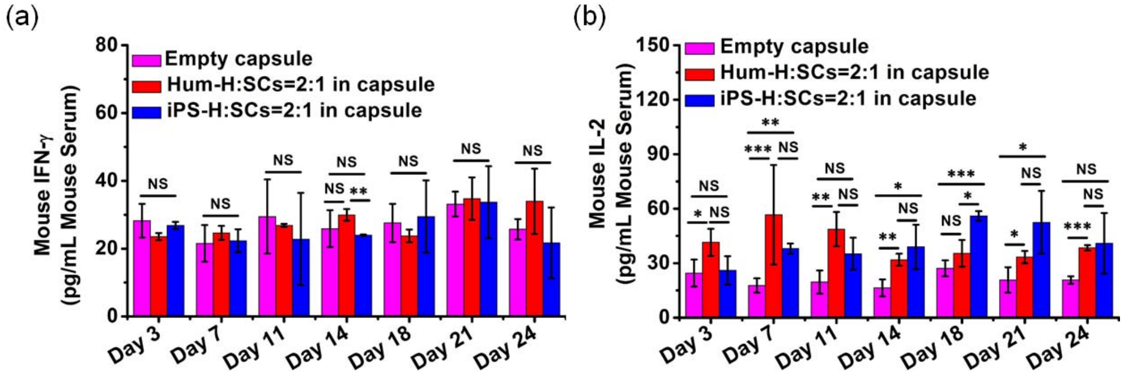

Fig. S6. The concentration of IFN-γ (a) and IL-2 (b) in mouse serum of empty capsules, Hum-H/SCs, and iPS-H/SCs transplanted mice. Mean ± SD (n = 3). **p* < 0.05, ***p* < 0.01, ****p* < 0.001, NS: no significant difference.

References

[1] Seglen PO. Preparation of isolated rat liver cells. In: Prescott DM, editor. Methods in cell biology. Academic Press, 1976. p. 29-83
